# Proteomics Analysis of Co-Purifying Cellular Proteins Associated with rAAV Vectors

**DOI:** 10.1371/journal.pone.0086453

**Published:** 2014-02-03

**Authors:** Biao Dong, Xunbao Duan, Hoi Yee Chow, Lingxia Chen, Hui Lu, Wenman Wu, Bernd Hauck, Fraser Wright, Philipp Kapranov, Weidong Xiao

**Affiliations:** 1 Department of Microbiology and Immunology, Sol Sherry Thrombosis Research Center, Temple University, Philadelphia, Pennsylvania, United States of America; 2 Cancer Biology Program, Fox Chase Cancer Center, Philadelphia, Pennsylvania, United States of America; 3 Department of Pathology and Laboratory Medicine, University of Pennsylvania School of Medicine, and Center for Cellular and Molecular Therapeutics, Children's Hospital of Philadelphia, Philadelphia, Pennsylvania, United States of America; 4 St. Laurent Institute, Cambridge, Massachusetts, United States of America; University of Kansas Medical Center, United States of America

## Abstract

Recombinant adeno-associated vectors (rAAV) are commonly purified by either chromatography or equilibrium CsCl gradient. Nevertheless, even after purification various cellular proteins often associate with rAAV vector capsids. Such co-purifying cellular proteins may raise concern about safety of gene therapy. Here we report identification and characterization of the co-purifying cellular protein in the vector preparations by using a combination of two proteomics approaches, GeLC-MS (gel electrophoresis liquid chromatography-mass spectrometry) and 2DE (two-dimensional gel electrophoresis). Most prominent bands revealed by Coomassie Blue staining were mostly similar to the AAV capsid proteins. Posttranslational modifications of capsid proteins were detected by the proteomics analysis. A total of 13 cellular proteins were identified in the rAAV vectors purified by two rounds of cesium chloride gradient centrifugation, including 9 by the GeLC-MS analysis and 4 by the 2DE analysis. Selected cellular proteins were verified by western blot. Furthermore, the cellular proteins could be consistently found associated with different AAV serotypes and carrying different transgenes. Yet, the proteins were not integral components of the viral capsis since a stringent washing procedure by column purification could remove them. These co-purified proteins in AAV vector preparations may have a role in various stages of the AAV life cycle.

## Introduction

Adeno-associated virus (AAV) is a nonpathogenic, replication-deficient parvovirus. It contains a single-stranded DNA genome of 4.7 kb, where two open reading frames (ORFs), Rep and Cap, are flanked by two inverted terminal repeats (ITR). Transcription initiation from internal promoters followed by splicing of the RNA transcripts generates four Rep proteins (Rep78, Rep68, Rep52 and Rep40) and three Cap proteins (VP1, VP2 and VP3) that assemble the virus particle at a ratio of 1∶1∶10. Up to now, more than 100 serotypes of AAV have been identified including the most studied serotype AAV2 [1].

Recombinant AAV (rAAV) has been used in clinical trials for a decade and is believed to represent one of the most promising vectors for gene therapy [2]–[5]. Currently, triple plasmid transfection is the most popular method to make rAAV vectors in which three individual plasmids supply the ITR-flanked transgene, the Ad-helper genes, and Rep/Cap genes [6]. Afterwards, the rAAV vectors can be purified by two successive rounds of CsCl gradient centrifugation to levels sufficient for most pre-clinical studies [7], [8]. To reach higher purity, the vectors can be further refined by chromatography techniques [8], [9]. Contaminants in the vector preparations, such as plasmid DNA, replication-competent AAV, and co-purifying cellular proteins, could decrease the efficiency of gene therapy and raise concerns about its safety [10]–[12].

On the other hand, knowledge of the co-purifying proteins can also improve our knowledge of cellular factors involved in viral biology and potentially lead to better vectors and production systems. Protein identification has benefited from advancements in sensitivity and accuracy of mass spectrometry (MS) that led to its broad use in proteomics [13]–[15]. GeLC-MS and 2DE are two popular approaches in proteomics due to the robust nature of the conventional SDS-PAGE and the resolving power of LC-MS/MS. To take full advantage of each method, we used a combination of both to detect cellular proteins in the rAAV vector preparations.

## Results

### Identification of proteins associated with rAAV vector by GeLC-MS

**Figure 1:**
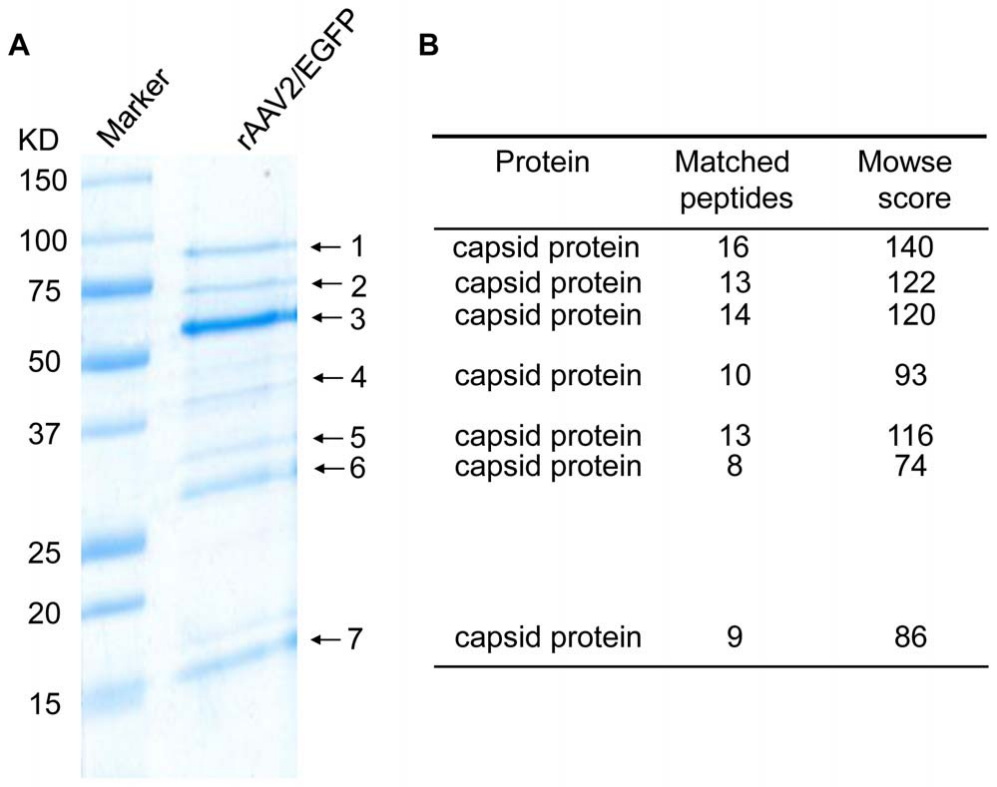
Identification of celluar proteins co-purifed with recombinant AAV vector. A, 1DE-SDS PAGE of recombinant AAV vector. The seven marked bands were excised and processed for MALDI-TOF analysis. B, All seven bands were identified as capsid-related proteins by the sequence homology. The Mowse score and the number of peptides matched were from the matching results against capsid protein VP1.

The AAV vector, AAV2-dsEGFP, was produced by triple plasmid transfection and purified by two-rounds of CsCl gradient centrifugation [16]. After 1D SDS/PAGE gel electrophoresis, Coomassie Blue staining of the gel revealed major protein components of the preparation (Fig 1). The AAV capsid proteins, VP1, VP2 and VP3, were detected as three major bands (band 1–3). In addition, several other bands with lower molecular weights than VP3, such as bands 4–7, were also observed. To search the identity of these proteins, bands 4–7 were excised out individually and analyzed by MALDI-TOF. A PMF database search suggested that all these proteins were capsid-related proteins with high confidence. Figure S1 shows the protein fingerprint of the band 7 as an example of MALDI-TOF identification. Of note, posttranslational modifications of protein, carbamidomethyl of cysteine and oxidation of methionine, were detected in two fragments in this analysis (Figure S1).

For the GeLC-MS analysis, the capsid protein components were resolved on 1D SDS/PAGE gel, stained with Coomassie Blue, and sliced to three parts according to the molecular weight, namely, >62 kD, 30–62 kD and <30 kD (Fig. 1). The proteins in the sliced gel fragments were then processed for MS/MS identification. Again, the major bands are AAV capsid related. Figure S2 shows the protein fingerprint of the band 7 as an example of MS/MS identification. In addition to viral capsid proteins, nine cellular proteins were detected and identified, which were showed in Table 1. Specifically, nucleolin, protein SET and splicing factor arginine/serine-rich 1 were identified in two fractions with different molecular weights, suggesting the alternatively spliced and/or modified forms of these proteins have strong affinity for the AAV capsids.

**Table 1:** List of cellular proteins identified using GeLC-MS analysis of adeno-associated virus vectors.

Molecular Weight	Swiss-Prot Accession #	Protein Name	Mowse Score	Peptides detected
>62 KD	P19338	Nucleolin	101	4
	P11279	Lysosome-associated membrane glycoprotein 1	82	2
	O00592	Podocalyxin-like protein 1	67	4
30–62 KD	Q01105	Protein SET	377	19
	Q07955	Splicing factor, arginine/serine-rich 1(SF2)	244	11
	P19338	Nucleolin	174	5
	P39687	Acidic leucine-rich nuclear phosphoprotein 32 family member A	90	2
	Q08170	Splicing factor, arginine/serine-rich 4	78	2
<30 KD	P06748	Nucleophosmin	185	12
	Q01105	Protein SET	149	10
	Q04837	Single-stranded DNA-binding protein, mitochondrial	144	4
	Q07955	Splicing factor, arginine/serine-rich 1(SF2)	135	5

### Identification of proteins associated with rAAV vector by 2-DE

**Figure 2:**
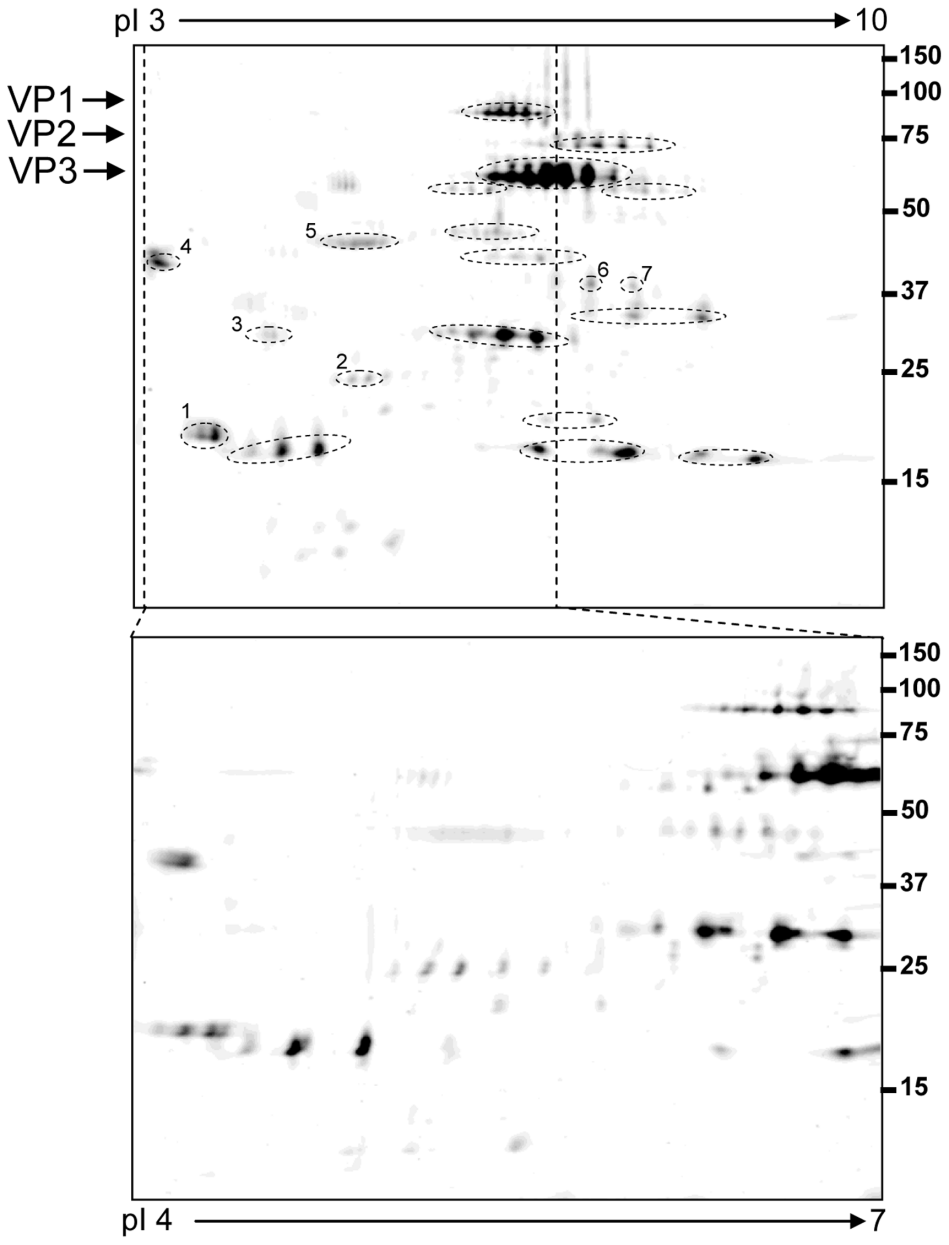
2DE map of recombinant AAV vector. The top 2DE map was obtained using a wide pH range of IPG strip pH–10, and the bottom one using a narrow pH range of IPG strip pH 4–7. The circled and numbered spots were identified as human proteins (Table 2), the spots only circled were identified as AAV capsid proteins.

As a complementary approach, 2DE was used to identify vector-associated proteins potentially missed by GeLC-MS. Figure 2 shows an example of protein distribution in a range of isoelectric point (IP) 3–10. The marked protein spots were extracted and processed for MS analysis. Seven different proteins were identified, including four cellular proteins that were missed by GeLC-MS (Table 2). Interestingly, distribution of each capsid protein as a series of distinct spots with similar molecular weights but different isoelectric points suggested presence of post-translational modifications in these proteins.

**Table 2:** List of human cellular proteins identified from 2DE analysis via nano-LC IT MS.

Spot	Swiss-Prot Accession#	Protein Name	Mowse Score	Peptides detected
1	P06748	Nucleophosmin	195	4
2	Q01105	Protein SET	379	9
3	P63104	14-3-3 protein zeta/delta	243	4
4	Q01105	Protein SET	436	11
5	P60709	Actin, cytoplasmic 1	308	8
6	P07355	Annexin A2	308	6
7	P04083	Annexin A1	159	2

### Confirmation of the proteins identified in rAAV vectors

**Figure 3:**
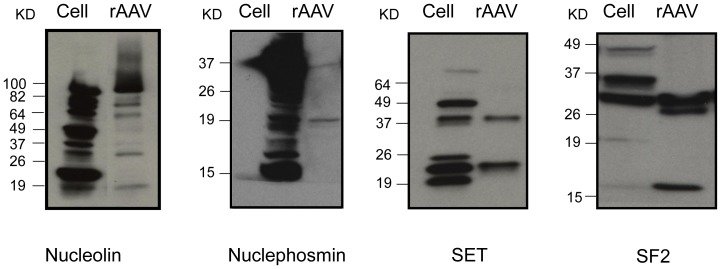
Western blot analysis of co-purified proteins in 293 un-infected cell lysates and purified AAV2-dsEGFP vector particles. 10×10^10^ viral particles were used for the western blot.

Western blotting was used to confirm the identities of the co-purified proteins in the rAAV vector with 293 cell lysates as the positive control. All four selected proteins detected in the AAV vector preparation could be detected in the 293 cell lysates, suggesting these proteins came from 293 cells during rAAV purification (Fig 3).

**Figure 4:**
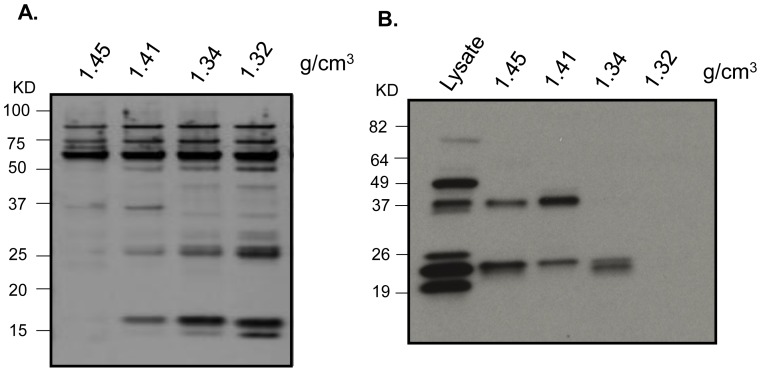
Detection of protein SET in different fractions of the AAV2-dsEGFP preparation. The vector was produced by triple plasmid transfection and purified by two rounds of cesium chloride ultra-centrifugation. The fractions with different densities were collected after the second round of ultra-centrifugation. Protein form 1×10^10^ viral particles was resolved on 10% SDS/PAGE. The full AAV vector particles have buoyant densities in CsCl from 1.41 to 1.45 g/cm^3^ while empty particles have the density of 1.32 g/cm^3^. A, silver staining; and B, western blot.

During rAAV purification, empty and full virus particles are separated according to their buoyant densities, 1.32 g/cm^3^ and 1.41 g/cm^3^, respectively. To test whether these co-purifying proteins distinguish between the types of viral particles, a range of different fractions during purification were used for western blotting by using antibodies against SET as an example. Equal amount of virus particles from each fraction were loaded in each lane as confirmed by silver staining of capsid proteins (Fig 4A). The results showed that SET was detected in the fractions containing full particles, but not in the fraction containing empty particles (Fig 4B).

**Figure 5:**
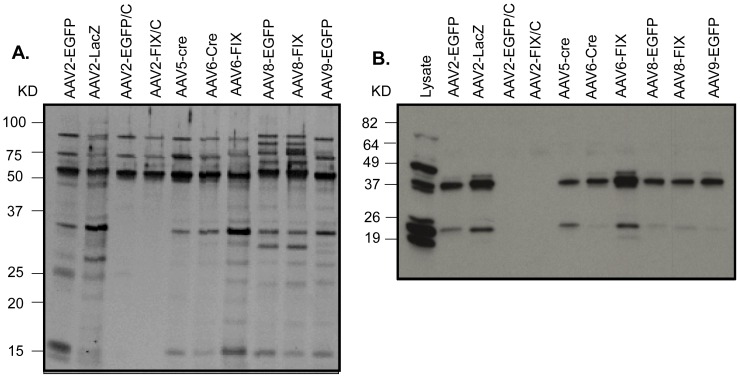
The effect of AAV serotypes, transgenes and purification methods on the presence of SET proteins in the rAAV vector preparations. Protein from 1×1010 viral particles was resolved on 10% SDS/PAGE. AAV2-EGFP/C and AAV2-FIX/C were purified by ion exchange chromatography. A, silver staining; and B, western blot.

To find other factors affecting the co-purifying cellular proteins of AAV vector, rAAV vectors prepared from different virus serotypes, carrying different transgenes and produced by different purification schemes were tested. Amount of protein from all vectors was confirmed by silver staining (Fig 5A) before the western blotting (Fig 5B). Three findings were revealed by the western blotting with the anti-SET antibody. First, the presence of this co-purified protein is serotype independent. The vector serotypes included the most popular ones 2, 5, 6, 8 and 9 and the protein could be detected in all of them. Second, its presence was transgene independent: the protein could be identified in the AAV vectors containing three different transgenes, EGFP, Cre and FIX. Third, the presence of the protein was dependent on the vector purification procedures. Vectors highly purified by chromatography (AAV2-EGFP/C and AAV2-FIX/C) did not contain the protein, unlike the same kind of vectors prepared by the CsCl gradient purification (Fig 5).

## Discussion

The purity is a major criterion for an AAV vector preparation used for human gene therapy in clinical applications. It is also conceivable that many cellular proteins take part in the AAV life cycle. In this study, we use two highly sensitive mass spectrometry approaches to identify viral and cellular proteins that are associated in the rAAV vector purified by CsCl gradient centrifugation. By using GeLC-MS and 2DE, we demonstrated for the first time that major bands smaller than VP3 are polypeptides which share similar protein fingerprints with the AAV capsid proteins. It suggests that those AAV related capsid fragments have a high affinity to the capsid proteins (Figure 1) and may contribute to the immunogenicity but not transgene expression.

Interestingly, a combination of GeLC-MS and 2DE identified thirteen cellular proteins (Table 1 and 2), including nucleolin [17], [18] and nucleophosm [17], [19] which have been reported to bind to the capsid during AAV packaging or infection process. However, the remaining 11 appear to represent novel cellular AAV capsid-binding proteins as evidenced by one such protein SET. The latter could be consistently detected in multiple batches of rAAV vectors containing different transgenes and based on various serotypes unlike other cellular proteins that randomly appeared in different vector fractions. It is possible that these proteins could bind to the common domains shared by the serotype capsids.

Function wise, some of these proteins may be involved in endosomal processing and nuclear transportation of the vector since packaged vector released from cells can re-infect other cells and continue normal vector transduction pathways. It is known that the rAAV capsid structure undergoes modifications in the endosomal processing and nuclear transportation, and some kinases such as PI3 kinase family play important roles in these processes [20], [21]. In this study, we identified 14-3-3 protein, which may interact with the kinases RAF1 and AKT1, two well-known downstream regulator of PI3 kinase. Of note, connection of several identified cellular proteins to protein phosphorylation suggests that phosphorylation of viral capsid proteins or cellular factors could be important for the vector packaging. Phosphorylation of capsid proteins has been reported to play a role be in the infection of rAAV particles [22]–[25]. In addition, specific association of SET polypeptide with full particles but not the empty ones is especially interesting and further suggests a role in packaging. Additional in-depth studies will be necessary to confirm this hypothesis.

Interestingly, “dirty” vectors may actually lead to better transduction performance [26]. Identification of SET protein may provide hypothetical support for such notion. This protein inhibits acetylation of nucleosomes and has a role in enhancing DNA replication of the adenovirus genome. It has two isoforms which are complexed with adenovirus viral core proteins. In addition, SET protein is a potent inhibitors of protein phosphatase 2A and considering the importance of phosphorylation in AAV transduction performance, it is tempting to hypothesize that this co-purifying protein has an intriguing role related in the AAV life cycle.

Confirmation of four selected cellular proteins by western blot suggests that the detection of co-purifying cellular proteins by proteomics analysis could be a fast, reliable methodology to control the quality of AAV vectors. Furthermore, using protein SET as an example, the presence of the cellular proteins is independent of transgenes and AAV serotypes, but dependent on the purification procedures. In this respect, the absence of the co-purified proteins from column-purified AAV vectors suggests that these proteins are not integral components of the AAV capsids. Also, since we do not have additional direct evidence to prove that proteins physically bind to AAV capsids, there is a possibility that these co-purifying proteins represent contaminants “co-sedimenting” or “co-migrating” with viral capsids after the regular 2 rounds of CsCl -purification. However, they do not appear as mere non-specific contaminants either because of the following reasons. First, we are not aware of any protein with a buoyant density of 1.4 g/ml representing the heavy viral particle with high DNA contents. Second, the association of the proteins with the capsids survived two rounds of CsCl-purification. This is a very strong indication that the proteins interact with the capsid. Third, at this stringency, we could identify only 13 such AAV-associated proteins out of thousands of cellular proteins. Of those, two are known and characterized cellular factors that play a role in the AAV life cycle. All this makes it likely that the novel co-purifying proteins could also function in the AAV biology and their exact roles warrant further characterization.

## Materials and Methods

### Production of AAV vector

A triple plasmid co-transfection method was used to produce the rAAV vectors used in this study (9). Briefly, one vector plasmid, one AAV helper plasmid, and one mini adenovirus function helper plasmid pFΔ6, were co-transfected into HEK293 cells cultured in roller bottles at a ratio of 1∶1∶2. The transfected cells were harvested 3 days later. rAAVs were then purified by two rounds of cesium chloride–gradient ultracentrifuge. After extensive buffer exchange against phosphate-buffered saline with 5% D-sorbitol, the peak fractions of purified virus were pooled and stored at −80°C before administration. Two rAAV vectors purified by ion exchange chromatography: AAV2-FIX were produced by GMP facility, Children's Hospital of Philadelphia (Philadelphia, PA) and AAV2-EGFP were kindly provided by Dr. Haifeng Chen, Virovek, Inc. (Hayward, CA).

### 1-DE Separation

10^12^ virus particles of AAV2-dsEGFP was solubilized in Tris-buffered saline solution (pH 8.0) containing 1% SDS. The protein concentration was then measured with Bradford-based BioRad protein assay using BSA as standard. 20 µg proteins were diluted with Laemmli sample buffer (BioRad) containing 5% β-mercaptoethanol. The mixture was heated for 10 min at 90°C and loaded onto 10–14% polyacrylamide gel. The separation was performed using a mini Protean II system (BiorRad) at 200 V for 45 min. The resolved protein bands were visualized with SimplyBlue SafeStain (Invitrogen).

### 2-DE Gel

2-DE was performed as described previously [27]. The proteins solubilized in TBS containing 1% SDS were precipitated with 80% acetone at −20°C overnight. The protein pellet after centrifugation was washed three times with 80% acetone to minimize SDS residue, and then solubilized in a buffer consisting of 7 M urea, 2 M thiourea, 40 mM Tris, and 65 mM DTT. Total 40 µg of sample protein was diluted in 125 µl with rehydration buffer and loaded onto an immobilized pH gradient strip (pH 3–10) by overnight passive in-gel rehydration. The rehydration buffer contains 8 M urea, 2% CHAPS, 0.2% carrier ampholytes and 10 mM DTT. Isoelectric focusing (IEF) within the strips was performed at 20°C with a MultiPhor II system (Amersham Biosciences Corp., Piscataway, NJ) using a total of 12,000 V h with a maximum of 4,000 V.

For 2nd dimensional separation, the IPG strips removed from the MultiPhor II chamber were reduced in 10 ml of an equilibration buffer (8 M urea, 30% glycerol, 2% SDS, 1% DTT, and 0.05 M pH 8.8 Tris) for 10 min, alkylated in 10 ml of a second equilibration buffer (with 2.5% iodoacetamide substituted for 1% DTT) for 15 min, and positioned against 10–14% SDS polyacrylamide gels in a BioRad Mini-PROTEAN® 3 System at 200 V for 45 min. Polyacrylamide gels were then fixed twice, each time for 30 min using 50% methanol, 7% acetic acid, balance water. Protein spots in the gels were revealed by staining with Sypro-Ruby fluorescence total protein stain. Fluorescence images of the gels were captured with FLA-5000 Fluor Imager (Fuji Photo Film Co, Ltd., Tokyo, Japan) and saved in TIFF format.

### In-Gel Trypsin Digestion

The visualized protein spots, protein bands, or gel sections were excised and then sliced into ∼1 mm gel cubes. Destaining of the sliced gel pieces was performed by two 40 min washes with 50% acetonitrile containing 50 mM ammonium bicarbonate. Following dehydration with 100% acetonitrile, 10 µl 12.5 ng/µl sequencing grade trypsin (Promega, Madison, WI) in 50 mM ammonium bicarbonate was added to the protein spot excised from 2DE gel, and 30 µl 12.5 ng/µl trypsin was added to gel band or gel section. The gel pieces with trypsin buffer were incubated overnight at 37°C. Resulting tryptic peptides were extracted twice with 15 µl extraction buffer (5% formic acid, 50% acetonitrile, balance water) for 20 min and the pooled extracts were processed with desalting ZipTips according to the manufacturer's instructions.

### MALDI-TOF- MS Analysis

The desalted peptides from protein band or 2DE spot were mixed 1∶1 with matrix solution (1% α-cyano-4-hydroxy cinnamic acid in 50% acetonitrile and 50% 0.1% trifluoroacetic acid) and 1.0 µl volumes were applied to wells of an AnchorChip™ sample target plate used for the Bruker Auto-flex MALDI-TOF-TOF instrument. Peptide mass fingerprints were obtained using the reflective and positive ion mode. Mass spectra were collected from the sum of 100–400 laser shots and mono-isotopic peaks were generated by FlexAnalysis™ software with signal-to-noise ratio of 2∶1. Mass peak value calculations used two trypsin auto-digestion peptides with M+H values 842.509 and 2211.104 as internal standards. Protein were identified by matching the calibrated peptide mass values within NCBInr protein databases using an in-house version of Mascot Server 2.2 imbedded in Bruker's Biotool software. Species viruses or homo sapiens was selected for taxonomy. Match variances allowed were a mass tolerance of 40 ppm, one missed trypsin cleavage, fixed modification of carbamidomethyl cysteine, and variable modification of methionine oxidation.

### Nano-LC-ESI-IT MS Analysis

*Nano-LC-ESI-IT MS* was carried out as described previously [28]. The desalted peptides were dried in a vacuum centrifuge and re-solubilized in 20 µl of 0.1% (vol/vol) trifluoroacetic acid. The tryptic peptide sample was loaded onto a 2 µg capacity peptide trap (CapTrap; Michrom Bioresources) separated by a C18 capillary column (15 cm 75 µm, Agilent) at 300 nl/min delivered by an Agilent 1100 LC pump. A mobile-phase gradient was run using mobile phase A (1% acetonitrile/0.1% formic acid) and B (80% acetonitrile/0.1% formic acid) from 0 to 10 min with 0–15% B followed by 10–50 min with 15–60% B and 50–55 min with 60–100% B. ESI was delivered using end-coated spray Silica tip (ID 20 µM, tip inner ID 10 µM, New Objective) at a spray voltage of −1300 V. Using an automatic switching between MS and MS/MS modes, MS/MS fragmentation was performed on the two most abundant ions on each spectrum using collision –induced dissociation. The complete system is fully controlled by HyStar 3.1 software. Mass spectra processing was performed with Data Analysis 3.3 and generated peak lists were submitted to an in-house Mascot server 2.2. The search was carried out against NCBInr database. Mascot search parameter are set as follows: species viruses or homo sapiens as taxonomy, trypsin enzyme with maximal 1 missed cleavage, cysteine carboxymethylation as fixed modification, methionine oxidation as variable modification, and 0.30 Da mass tolerance for MS and 0.4 Da for MS/MS. A protein was considered to be positively identified if at least two different peptides with ion scores 33 or higher (95% confidence threshold) in the Mascot MS/MS ion search report.

### Silver staining

Purified virus (∼1×10^10^ particles) was mixed with protein loading dye and heated at 95°C for 5 minutes. The denatured samples were then separated on 10% SDS-PAGE gel. After electrophoresis, the gel was stained by Pierce® Silver Stain Kit (Thermo Scientific, Rockford, IL) according to the manufacturer's instruction.

### Western blot

rAAV vectors (∼1×10^10^ particles) were mixed with protein loading dye and heated at 95°C for 5 minutes. The denatured samples were then separated on 10% SDS-PAGE gel and transferred to nitrocellulose membrane (Bio-Rad, Hercules, CA). After blocking the membrane with 5% nonfat dry milk in TBST buffer (25 mM Tris⋅HCl at pH 8.0, 150 mM NaCl and 0.1% Tween 20), the membranes were incubated with the primary antibodies, Nucleolin (Abcam, Cambridge, MA, 1: 1,000), Nucleophosmin (Abcam, Cambridge, MA, 1∶500), SET(I2PPA E-15, Santa Cruz, CA, 1∶200) or SF2 (Invitrogen, Carlsbad, CA, 1∶500), at 4°C overnight. The membrane was washed and incubated with the second antibodies, a HRP-conjugated sheep anti-mouse IgG antibody (Sigma, St Louis, MI, 1∶2,000) or anti-rabbit (Abcam, Cambridge, MA, 1∶3,000). The membrane was developed using an enhanced chemiluminescent substrate (Amersham-Pharmacia Biotech, Piscataway, NJ).

## Supporting Information

Figure S1 **Identification of Band 7 as capsid protein by MALDI-TOF analysis.** The peptide mass fingerprinting was internally calibrated with autotryptic peaks, resulting in high mass accuracy and high Mowse score. The search results showed that 8 matched peptides were from two different regions within the sequence of capsid protein VP1.
(TIF)

Figure S2 **Identification of Band 7 as capsid protein VP1 by nano-LC-IT MS.** Ten unique peptides were identified based on sequence information with a cumulative Mowse score of 601. The left panel lists all the peptides identified including the location of each peptide. The right panel is an example of an MS/MS spectrum for one peptide K.RWNPEIQYTSNYNK.S. The matched y ions and b ions are labeled. It should be noted that these 10 peptides are in the two different regions of the protein sequence similar to the peptides obtained from MALDI-TOF analysis.
(TIF)
