# Supplementary figures and images for: Proteomics Analysis of Co-Purifying Cellular Proteins Associated with rAAV Vectors
Source: PLoS One. 2014 Feb 3;9(2):e86453. doi: 10.1371/journal.pone.0086453 (PMC3911921; doi:10.1371/journal.pone.0086453)

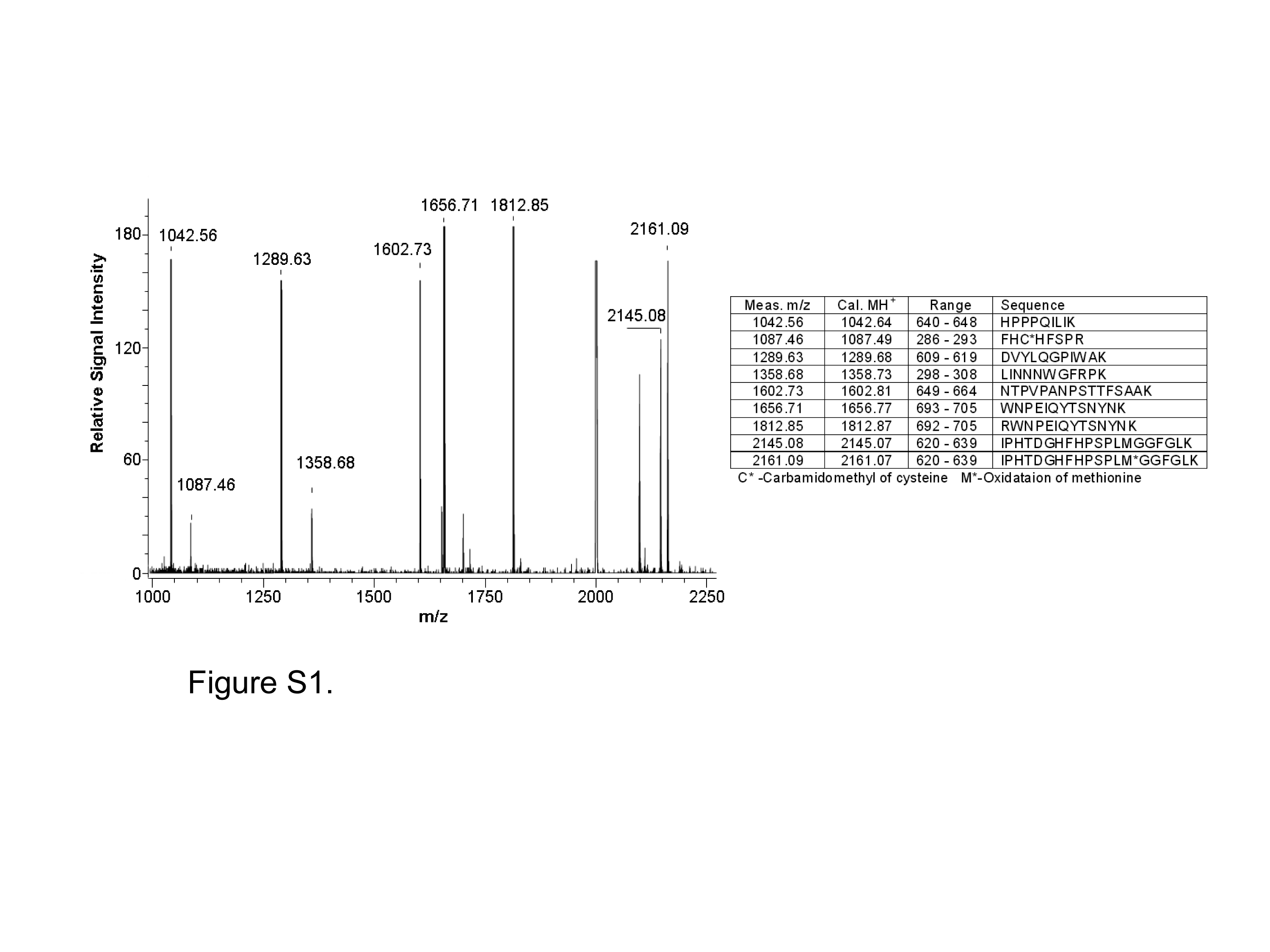

Supplement: Figure S1 — Identification of Band 7 as capsid protein by MALDI-TOF analysis. The peptide mass fingerprinting was internally calibrated with autotryptic peaks, resulting in high mass accuracy and high Mowse score. The search results showed that 8 matched peptides were from two different regions within the sequence of capsid protein VP1. (TIF) [file pone.0086453.s001.tif]

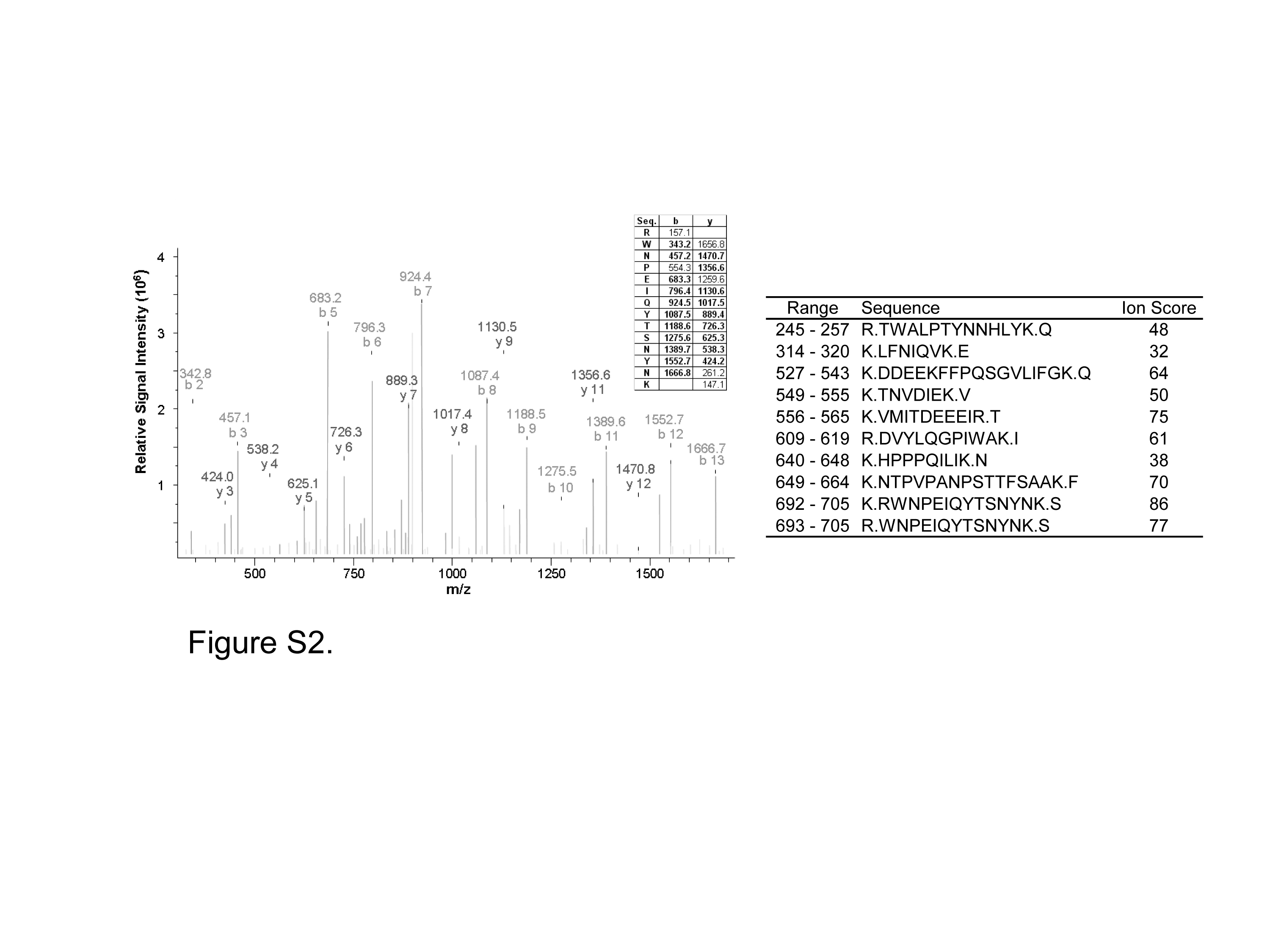

Supplement: Figure S2 — Identification of Band 7 as capsid protein VP1 by nano-LC-IT MS. Ten unique peptides were identified based on sequence information with a cumulative Mowse score of 601. The left panel lists all the peptides identified including the location of each peptide. The right panel is an example of an MS/MS spectrum for one peptide K.RWNPEIQYTSNYNK.S. The matched y ions and b ions are labeled. It should be noted that these 10 peptides are in the two different regions of the protein sequence similar to the peptides obtained from MALDI-TOF analysis. (TIF) [file pone.0086453.s002.tif]
